# Supplementary material for: The genome sequence of an ichneumonid wasp, Polytribax perspicillator (Gravenhorst, 1807)
Source: Wellcome Open Res. 2025 Mar 18;10:140. [Version 1] doi: 10.12688/wellcomeopenres.23769.1 (PMC12015431; doi:10.12688/wellcomeopenres.23769.1)
Supplement: Peer review history

# Reviewer report: Reviewer response for version 1

**DOI:** 10.21956/wellcomeopenres.26217.r121935

**Published:** 2025-04-24

**Authors:** Carl E Hjelmen

**Affiliations:** 1 Utah Valley University, Utah, USA

In this manuscript the authors present the genome sequence and assembly of an ichneumonid wasp ( *Polytribax perspicillator*). The authors utilize PacBio HiFi and Hi-C methodologies seen often in projects from the Tree of Life project. The authors do a great job giving background to the organism and why it would be interesting to sequence and assemble. This species is commonly collected and observed, as well as being parasitoids.

 The methodologies, including collection and storage, are detailed enough to be repeatable, and the assembly assessments seem to suggest a quality assembly.  I really appreciate the table of software tools and the version of the software being used in this study. This adds a lot to the repeatability of this work. All data appears to be publicly available as well.

 Issues:

 In the “sequencing data” section, the authors base their coverage based on the genome size estimation from assembly.  Assembly based methods for estimating genome size is known to have issues.  Have you compared this genome size estimation to others in the genus/family or estimated this species genome size?

 This mitochondrial genome assembly size (25.68 kb) seems quite large; usually, I’d expect to see something between 15-20kb. Have you aligned this with other wasp mitochondria to see how they compare?

 The axes on the Blobplot seem a little strange, particularly the horizontal axis (gc content).  The plot would benefit from expanding the axis to show more of the data.

Are sufficient details of methods and materials provided to allow replication by others?

Yes

Is the rationale for creating the dataset(s) clearly described?

Yes

Are the datasets clearly presented in a useable and accessible format?

Yes

Are the protocols appropriate and is the work technically sound?

Yes

Reviewer Expertise:

genomics, entomology, evolution, phylogenetics

I confirm that I have read this submission and believe that I have an appropriate level of expertise to confirm that it is of an acceptable scientific standard.

# Reviewer report: Reviewer response for version 1

**DOI:** 10.21956/wellcomeopenres.26217.r121936

**Published:** 2025-04-22

**Authors:** Alejandro Zaldívar Riverón

**Affiliations:** 1 Universidad Nacional Autónoma de México, Ciudad de Mexico, Mexico

This study presents the chromosomally complete genome sequence of the ichneumonid wasp *Polytribax perspicillator, a species collected in the U.K.* The report follows the same format as previous genome sequences published by the authors. The information is complete and appropriate, and I recommend it for indexing without corrections.

Are sufficient details of methods and materials provided to allow replication by others?

Yes

Is the rationale for creating the dataset(s) clearly described?

Yes

Are the datasets clearly presented in a useable and accessible format?

Yes

Are the protocols appropriate and is the work technically sound?

Yes

Reviewer Expertise:

Entomology, systematics

I confirm that I have read this submission and believe that I have an appropriate level of expertise to confirm that it is of an acceptable scientific standard.

## References

[1] : The genome sequence of an ichneumonid wasp, Exephanes ischioxanthus (Gravenhorst, 1829). *Wellcome Open Res* .2024;9: 10.12688/wellcomeopenres.20498.1 8 10.12688/wellcomeopenres.20498.1 39233900 PMC11372355

# Reviewer report: Reviewer response for version 1

**DOI:** 10.21956/wellcomeopenres.26217.r120990

**Published:** 2025-04-03

**Authors:** Taro Nakamura

**Affiliations:** 1 National Institute for Basic Biology, Okazaki, Japan

**General comments:**

 This manuscript presents a chromosomal-level genome assembly of the ichneumonid wasp *Polytribax perspicillator* using advanced sequencing technologies, including PacBio HiFi sequencing and Hi-C chromosomal scaffolding. The assembly quality is high, clearly meeting current genomic standards, and detailed methodological transparency ensures reproducibility.

**Specific Comments:**

 Strengths:

1. High-quality genome assembly: The authors effectively leveraged PacBio HiFi sequencing and Hi-C data, achieving an exceptionally complete chromosomal-level assembly (eight chromosomal pseudomolecules). Assembly statistics and BUSCO completeness (96%) strongly support the high quality of the generated genome.
2. Clear and reproducible methodology: The methods section is comprehensive and transparent, offering sufficient detail for reproducibility. Procedures for sample collection, nucleic acid extraction, sequencing, assembly, and quality control are well-described.
3. Rigorous quality assessment: The genome assembly quality was thoroughly assessed using multiple standards (e.g., BUSCO completeness, Merqury QV), ensuring robustness and accuracy.

Are sufficient details of methods and materials provided to allow replication by others?

Yes

Is the rationale for creating the dataset(s) clearly described?

Yes

Are the datasets clearly presented in a useable and accessible format?

Yes

Are the protocols appropriate and is the work technically sound?

Yes

Reviewer Expertise:

Developmental biology, Evolutionary biology

I confirm that I have read this submission and believe that I have an appropriate level of expertise to confirm that it is of an acceptable scientific standard.
